# Assessment of Compliance With NICE Guidelines on Safety Planning Following Self-Harm in Elderly Patients in a Mental Health Trust

**DOI:** 10.1192/bjo.2024.637

**Published:** 2024-08-01

**Authors:** Safyan Tariq, Aparna Prasanna, Ryan Lau

**Affiliations:** Black Country Healthcare, Wolverhampton, United Kingdom

## Abstract

**Aims:**

Our aim was to evaluate the extent to which the risk assessment protocol post self-harm incidents for patients aged over 65 at the Black Country Healthcare Trust is aligned with the recommendations set forth in the NICE Guideline (NG225). We specifically sought to determine whether safety plans are incorporated as recommended by the NICE Guideline (NG225), and in the absence of a distinct safety plan, whether essential components of such a plan are integrated within the risk assessment framework utilised following episodes of self-harm.

**Methods:**

A retrospective audit was conducted utilizing data from the trust on self-harm incidents over a six-month duration. Of the 1,408 recorded incidents, 68 involved individuals aged 65 years or older. A sample of 30 incidents was randomly selected from this cohort to constitute the target sample for this study. Each case was anonymized with a unique identifier and subjected to a comprehensive review employing a bespoke data collection instrument, expressly developed for this audit. The review process was facilitated by the trust's digital record system (RIO). Data collated for analysis encompassed a range of variables, including demographic details, diagnostic classifications, geographical location, care setting, self-harm methodologies, the severity of the self-harm events, the origin of data, and compliance with the stipulated criteria of the NICE Guidance (NG225).

**Results:**

Comprehensive safety plans were present in a minority of cases, specifically 6.7% (2 out of 30 patients). The documentation of individual components of the safety plan, analysed separately, yielded the following results:

Documentation of self-harm mechanisms was achieved in 70% of cases (21/30).
2. Identification of precipitants or triggers was noted in 56.7% of cases (17/30).
3. The formulation of coping strategies was documented in 20% of the sample (6/30).
4. The enumeration of essential contacts was completed in 33.3% of cases (10/30).
5. The identification of family members pertinent to the patient’s support network was noted in 33.3% of cases (10/30).
6. The inclusion of contact details for these identified individuals was present in 30% of cases (9/30).
7. Guidelines to ensure a safe environment were applicable and recorded in 38.9% of the relevant cases (7/18).

**Conclusion:**

The majority of patients did not have a safety plan post self-harm incidents. Notwithstanding the absence of a comprehensive safety plan, critical elements prescribed by NICE Guidance (NG225) were insufficiently addressed within the risk assessment and subsequent management planning post self-harm.

